# Membrane Proteins: Structure, Function and Motion

**DOI:** 10.3390/ijms24010468

**Published:** 2022-12-27

**Authors:** Masoud Jelokhani-Niaraki

**Affiliations:** Department of Chemistry and Biochemistry, Wilfrid Laurier University, Waterloo, ON N2L 3C5, Canada; mjelokhani@wlu.ca

Cell membranes are intricate multicomponent supramolecular structures, with a complex variable morphology and chemical composition. Membrane proteins are present in the cell membranes of all living organisms and contribute to their biological and physicochemical properties. The structure, function and mobility of membrane proteins are closely intertwined with the structure and composition of membranes and their surrounding environment. Cell membranes are essential for the steady-state homeostasis of cells and their reaction to environmental changes. Membrane proteins are involved in a variety of dynamic cellular processes, such as ionic and molecular transport, electron transport, signal transduction, enzymatic reactions and intercellular communication. Despite their relative abundance (25–30% of all proteins) and important roles in life processes, there is considerably less structural and functional information about membrane proteins in comparison to other types of proteins. The first high-resolution structure for a membrane protein was published in 1985 [1,2]. Currently, more than 1500 unique membrane protein structures have been determined in atomic resolution [3]. In comparison, by the end of 2022, close to 200,000 protein structures have been deposited in the Protein Data Bank [4].

The main challenge in studying the native structures of membrane proteins and their biological functions is in optimizing the experimental conditions for their extraction/purification from cell membranes and successive reconstitution in their native-like membrane milieus [5]. In their native environments, membrane proteins interact with membrane lipids, other membrane proteins, as well as extramembranous water, inorganic and small organic ions and other biomolecules. All these interactions, the everchanging chemical composition of the cell and the extracellular environmental factors influence the structure and biological function of membrane proteins in a time-dependent dynamic system. These variable interactions and the complexity of cell membrane structures are not easily reproducible (or impossible to reproduce) experimentally under in vitro or modified in vivo conditions. In addition to arduous technical problems in isolation and purification of membrane proteins in their intact native conformations, there are many questions about the molecular behaviour of membrane proteins that are not yet answered or are only partially answered. Some of these problems include the specific and non-specific interactions of membrane proteins with lipids and other membrane or membrane-interacting proteins, dynamic conformational changes in membrane proteins, membrane protein folding and oligomerization, modes of action of infectious structures (such as viruses) with membrane surface and membrane proteins, role of membrane proteins in morphology of cell and organelle membrane structures and the molecular action and adaptability of membrane proteins in their complex in vivo environment. As a result of these substantially challenging problems, in the past few decades, molecular studies of membrane proteins have become an attractive and flourishing research area in biochemistry, molecular biophysics, cell biology and systems biology. Visualizing/imagining the dynamic nature of membrane proteins and their various interconnections in the cell is becoming one of the key elements in understanding the colourful, intricate and mysterious molecular machinery of life.

In this compendium of reviews and research articles on the structure, function and motion of membrane proteins, we can see diverse experimental, theoretical and computational approaches in studying different aspects of the biophysics and biochemistry of membrane proteins. Despite several important breakthroughs in deciphering the molecular aspects of membrane proteins and structure of membranes, this unusual diversity in approaches emphasizes the exploratory nature of the research in this field, which is still in its initial steps in a slightly illuminated but foggy territory.

At the beginning of this journey, the general reader can start from a brief but comprehensive review on the difficulties and uncertainties facing researchers in the current methods of extraction, purification and reconstitution of membrane proteins in native-like lipid surroundings, highlighting the advantages and disadvantages of each method [6]. Lipids, as the essential components in cell membranes, interact with embedded membrane proteins and influence their structure, function, association and conformational flexibility. Two insightful reviews emphasize the biophysical aspects of lipid–protein interacting systems and various computational and experimental methodologies currently used for studying these molecular systems [7,8].

The transport of ions, as well as small molecules and macromolecules, through membranes is closely connected to the membrane protein structure and function, structure of membranes and their chemical composition. A group of articles in this collection focuses on this aspect of membrane proteins. The mechanism of the transport of protons through the inner membrane of mitochondria by structurally connected carrier proteins adenine nucleotide translocase (ANT) and uncoupling protein (UCP) is considered in detail in a review and two articles. The detailed review introduces the current views on the connection between the structure and mechanism of proton transport in mitochondrial membrane proteins, UCP and ANT [9]. In a related article, it is hypothesized that the proton transport in ANT and UCPs has similar regulation patterns that can be explained by the fatty acid cycling concept [10]. A molecular dynamics (MD) simulation study, based on the homology modelling with ANT, shows that the UCP2 structure is impermeable to water and possesses additional functional elements, such as a specific fatty-acid-binding site, which is related to the proton transport mechanism across the inner-mitochondrial membranes [11]. Proton transport is also the subject of a review article on the connection and importance of cellular and subcellular protons on the mechanism of activity of some molecule transporters essential for the biological processes in plants [12]. In a biophysical study on reconstituted archaerhodopsin photoreceptor protein, a proton pump, the real-time pore-forming properties of this protein is studied in bilayers [13]. An inactivated non-conducting conformational state of KcsA (the bacterial potassium ion channel) with minimal affinity for potassium ions, while maintaining two binding sites for K^+^ ions, was detected by using a long-chain quaternary ammonium channel blocker [14].

Molecular transport and targeting mediated by the membrane proteins in cell and organelle membranes are the subject of a review and two articles. Recent advances in the relatively unexplored area of protein targeting and protein/molecular transport in the chloroplast outer membrane are reviewed with a focus on the characterized chloroplast outer-membrane protein targeting pathways and new insights into novel targeting pathways using a bioinformatics approach [15]. Bacterial resistance-nodulation-cell division (RND) membrane protein transporters are a group of efflux pumps involved in the mechanisms of bacterial resistance against antibiotic drugs. In a comparative approach, these inner-membrane proteins are studied in two Gram-negative bacteria, with a special stress on the role of the inter-membrane peptidoglycan layer in stabilizing the RND protein complexes that surpass the inter-membrane space to reach the outer-bacterial membrane [16]. Transepithelial water flux at the tricellular tight junction for angulin-1 (lipolysis-stimulated lipoprotein receptor, LSR) is studied in epithelial cell lines to conclude that the transepithelial water permeability was affected only in tight cell lines [17].

The specific influence of lipid-interacting proteins and peptides on the membrane structure and its physicochemical properties is treated in three articles. The amyloid β (Aβ) cascade hypothesis has been proposed as a molecular mechanism for the neurodegenerative Alzheimer’s disease (AD). Employing a combination of experimental and MD simulation methods, an alternative hypothesis is proposed for AD, based on the preservation of the mechanical balance in neuronal membranes, by investigating the effect of two Aβ peptides on membrane mechanical properties [18]. Tetraspanins are surface-active transmembrane proteins that form a network of protein–protein interactions within the plasma membrane, mediated by specific lipids. A single-molecule approach in mammary epithelial cells is used to study the membrane behavior and cell surface interconnected dynamics of tetraspanins, as well as the effect of gangliosides in the generation of tetraspanin-enriched areas [19]. It has been shown that small Heat-Shock Proteins (sHSPs) interact with lipids to modulate the physical state and integrity of cell membranes. Association with lipid membranes, strong preference of fluid membranes and the strong influence on the phase behaviour of plasma membranes are investigated for a less studied protein, HSPB1 [20].

Structure, function and dynamics of membrane and membrane-interacting proteins are the subject of one review and two computational articles. Membrane cytochrome b5 reductase is a single-electron oxidoreductase that facilitates the reduction of several biological acceptors in cellular membranes. Employing both computational and experimental methods of analysis of protein structures and dynamics, roles of amino acid and catalytic domains within cytochrome b5 reductase, as well as structural domains involved in cytochrome b5 interactions with other electron acceptors have been reviewed [21]. A kinetic model to be used for extensive simulations of the synaptic transmission process is challenging. In a systems biology study, a compartmentalized kinetic model for CA3-CA1 synaptic transmission is proposed to predict the functional impact caused by disease-associated variants of NMDA (N-Methyl-D-aspartic acid) receptors causing severe cognitive impairment [22]. In addition to focusing on the overall conformation and structural dynamics, microsecond MD simulations of UCP2 in the phospholipid bilayers show that ATP binding in the UCP2 cavity is tight and possesses a fatty-acid-binding site at the R60 region that is related to the proton transport mechanism across the inner-mitochondrial membrane [11].

The effects of interactions of infectious agents with cell membranes and membrane proteins, the cellular processes and cascades of events that follow, as well as their implications in the molecular mechanisms of infection and drug/vaccine development are the subject of one review and two articles. In a timely comprehensive survey of viral membrane proteins and their functional interactions and pathogenesis (with focus on SARS-CoV-2, or Severe Acute Respiratory Syndrome Coronavirus-2), the recent information on Coronavirus proteins and membrane proteins in the Coronaviridae family (including information on their structures, functions and participation in pathogenesis) is reviewed. In this review, some current CoV vaccine development strategies with purified proteins, attenuated viruses and DNA vaccines are also discussed [23]. Syk (Spleen tyrosine kinase) inhibitors can be considered as potential effective antimalarial drugs. This protein can be found in human erythrocytes targeting the membrane protein band 3. Tyr phosphorylation of band 3 occurs during the malaria parasite’s growth, weakening the host cell membrane, causing easier reinfection. It is shown that the presence of Syk inhibitors decrease band 3 Tyr phosphorylation with the increase in the antimalarial drug’s concentration [24]. Fusion of viral and host cell membranes is crucial in the life cycle of enveloped viruses. In influenza virus, this fusion is mediated by subunit 2 of hemagglutinin (HA) glycoprotein. MD simulations combined with experimental studies of three HAfp peptide variants are employed to characterize their free-energy landscape and interaction with the lipid bilayer. It is shown that the effect of deeply inserted peptides is significant in the membrane fusion process and correlates with the insertion depth of the N-terminal amino group [25].

Membrane proteins are involved in controlling/maintaining the integrity of cells, as well as defence and control mechanisms against programmed cell death. A review and two articles explore some examples of these functions of membrane proteins. FtsH proteins are membrane-bound ATP-dependent zinc metalloproteases essential in proteolysis of unneeded or damaged membrane proteins found in bacteria, animals and plants. In eukaryotic cells, their location is restricted to chloroplasts and mitochondria. Findings concerning the FtsHi pseudo-proteases and their involvement in protein import in the model organism *Arabidopsis thaliana* are reviewed [26]. A critical step in apoptosis is the permeabilization of the outer-mitochondrial membrane, controlled by Bcl-2 family proteins. Binding and conformational dynamics of two main Bcl-2 family members, the pore-forming protein Bax and the truncated form of the activator protein Bid (tBid) are imaged and characterized at the single-particle level in a mitochondria-like planar lipid bilayer [27]. Comparable to apoptosis, ferroptosis is programmed cell death mediated by iron-dependent lipid peroxidation. The role of the NO• radical on suppression of ferroptosis has been suggested. To further explore the molecular mechanism of this suppression, a biochemical model, combined with lipidomics and structure-based modeling and simulations, has been utilized. The results of this study offer original insights into the molecular mechanism of repression of a ferroptotic peroxylated phosphatidylethanolamine (PE) production by NO• [28].

Involvement of membrane proteins in cell and organelle membranes can induce dynamic morphological changes that influence the overall cell function. These morphological modifications are detectable through high-resolution microscopy and connected to molecular interactions of membrane proteins in their native milieus. The membrane domain of eukaryotic HMG-CoA reductase (HMGR: 3-hydroxy-3-methylglutaryl CoA reductase) induces endoplasmic reticulum (ER) proliferation and membrane association into Organized Smooth Endoplasmic Reticulum (OSER) structures. OSER structures grow via the incorporation of ER membranes on their periphery and progressive compaction to the inside. The ER-HMGR domains are highly dynamic and can act as active components of the eukaryotic cells [29].

Limited mutations in membrane proteins may enhance their functional expression in experimental systems, without a significant effect on their functional sensitivity. An example is olfactory receptors (ORs) in vertebrate animals. ORs are members of the G protein-coupled receptor (GPCR) family. To overcome technical difficulties in studying these proteins, their functional expression has been improved by single amino acid substitution at one of the two sites in the OR-conserved residues without causing alterations in the odorant responsiveness, implying that specific sites within transmembrane domains in some ORs can regulate their membrane expression [30].

In conclusion, I would like to thank all the contributors to this collection and hope that it can contribute and add to the breadth and depth of biophysical and biochemical research on molecular and cellular aspects of Membrane Proteins.
